# Vitamin D deficiency may predict a poorer outcome of IgA nephropathy

**DOI:** 10.1186/s12882-016-0378-4

**Published:** 2016-11-02

**Authors:** Xiao-Hua Li, Xin-Ping Huang, Ling Pan, Cheng-Yu Wang, Ju Qin, Feng-Wei Nong, Yu-Zhen Luo, Yue Wu, Yu-Ming Huang, Xi Peng, Zhen-Hua Yang, Yun-Hua Liao

**Affiliations:** Renal Division, Department of Medicine, First Affiliated Hospital of Guangxi Medical University, Nanning, 530021 Guangxi Zhuang Autonomous Region China

**Keywords:** Disease progression, IgA nephropathy, Prognosis, Risk factor, Vitamin D

## Abstract

**Background:**

Experimental studies showed that 25-hydroxy-vitamin D [25(OH)D] deficiency (defined as 25-hydroxy-vitamin D < 15 ng/ml) has been associated with CKD progression. Patients with IgA nephropathy have an exceptionally high rate of severe 25(OH)D deficiency; however, it is not known whether this deficiency is a risk factor for progression of IgA nephropathy. We conducted this study to investigate the relationship between the plasma level of 25(OH)D and certain clinical parameters and renal histologic lesions in the patients with IgA nephropathy, and to evaluate whether the 25(OH)D level could be a good prognostic marker for IgA nephropathy progression.

**Methods:**

A total of 105 patients with biopsy-proven IgA nephropathy were enrolled between 2012 and 2015. The circulating concentration of 25(OH)D was determined using serum samples collected at the time of biopsy. The primary clinical endpoint was the decline of estimated glomerular filtration rate (eGFR; a 30 % or more decline compared to the baseline).

**Results:**

Mean eGFR decreased and proteinuria worsened proportionally as circulating 25(OH)D decreased (*P* < 0.05). The 25(OH)D deficiency was correlated with a higher tubulointerstitial score by the Oxford classification (*P* = 0.008). The risk for reaching the primary endpoint was significantly higher in the patients with a 25(OH)D deficiency compared to those with a higher level of 25(OH)D (*P* = 0.001). As evaluated using the Cox proportional hazards model, 25(OH)D deficiency was found to be an independent risk factor for renal progression [HR 5.99, 95 % confidence intervals (CIs) 1.59–22.54, *P* = 0.008].

**Conclusion:**

A 25(OH)D deficiency at baseline is significantly correlated with poorer clinical outcomes and more sever renal pathological features, and low levels of 25(OH)D at baseline were strongly associated with increased risk of renal progression in IgAN.

**Electronic supplementary material:**

The online version of this article (doi:10.1186/s12882-016-0378-4) contains supplementary material, which is available to authorized users.

## Background

IgA nephropathy (IgAN) is the most common form of glomerulonephritis worldwide [1], especially in Asia, and represents one of the main causes of the end-stage renal diseases (ESRD) [2]. Male gender, early-onset, absence of macroscopic hematuria, persistent microscopic hematuria, hypertension, proteinuria, presence of renal dysfunction at the time of diagnosis, and certain histological features of renal lesions have been identified as important risk factors for its progression[3–6].

Recent observations suggest that low vitamin D levels, measured as 25-hydroxyvitamin D [25(OH)D] is significantly associated with a more severe decrease in estimated glomerular filtration rate (GFR) in patients with chronic kidney diseases (CKD) [7, 8]. A series of studies have also suggested a role of vitamin D [9] deficiency (defined as 25-hydroxy-vitamin D < 15 ng/ml) [10] in the short-life expectancy of CKD [11–13]. Bienaim’ et al. also reported that patients with lower serum total 25(OH)D concentrations at 3 months after transplantation exhibited lower kidney allograft function at 1 year after transplantation and had higher risk of the progression of interstitial fibrosis and tubular atrophy. [14] Furthermore, among patients with wide range of renal dysfunctions including ESRD, vitamin D deficiency showed the associations with vascular calcification, vascular endothelial function, cardiovascular events, and cardiovascular mortality. In the Third National Health and Nutrition Examination Survey (NHANES III) cohort, individuals with 25 (OH) vitamin D levels < 15 ng/ml had a higher risk for all-cause mortality despite adjustments for CKD stage and for potential confounders. [15] Therefore, low vitamin D level is considered as a candidate novel risk factor for poor outcome of renal disease. Experimental data have indicated that vitamin D analogues mediate a decrease in albuminuria and slow the progression of kidney injury in several animal models of kidney disease [16, 17]. Moreover, a randomized trial by Liu and colleagues [18] has demonstrated that oral calcitriol results in decrease in proteinuria in IgA nephropathy patients confirming earlier results of Szeto et al. [19]. Experimental studies have revealed that VD insufficiency upregulates the renin-angiotensin system [20, 21] and the NF-κB pathway [22], decreases the nitric oxide synthase transcription in vascular endothelial cells [23–25], increases inflammation and oxidative stress [26, 27], and therefore may be a risk factor for progression of kidney diseases. However, to date there have been few studies exploring the relationship between the 25(OH)D level and the progression of IgAN, we therefore studied this aspect.

## Methods

### Patients and serum samples

A total of 105 patients with newly diagnosed, biopsy-proven primary IgAN were recruited from The First Affiliated Hospital of Guangxi Medical University between 2012 and 2015. IgAN was diagnosed with mesangial depositions of IgA under immunofluorescence microscope and with electron-dense deposits in the mesangium under electron microscope. Blood samples were collected before initial VD treatment at the time of kidney biopsy.

### Basic data and study endpoint

Patient demographics as well as conventional clinical parameters including age, gender, body mass index (BMI), blood pressure (BP), blood chemistry and daily proteinuria were collected at the time of kidney biopsy. Blood chemistry tests included serum creatinine, albumin, uric acid, total cholesterol and IgA. Estimated glomerular filtration rate (eGFR) was calculated using the Chronic Kidney Disease Epidemiology Collaboration (CKD-EPI) equation [28]. Consecutive changes in renal function and the daily proteinuria were recorded during the follow-up period. Primary endpoint was defined as a decline of 30 % or more in eGFR compared with the baseline. Medication history was recorded, including the use of renin-angiotensin system (RAS) blockers such as angiotensin-converting enzyme inhibitors and angiotensin II receptor blockers, and immunosuppressors (IS) within 6 months of kidney biopsy and during the follow-up period. Kidney biopsy was performed to those patients with uncontrolled proteinuria or uncontrolled hypertension in spite of the use of RAS blockers. However, according to the clinical judgment of the nephrologists, it would often be performed regardless of the efficacy of RAS blockers. The patients who had a persistant proteinuria greater than 1 g after the treatment of RAS blocker or showed impaired renal function usually received immunosuppressive treatment for 2-4 weeks after the kidney biopsy. Steroid medicines were the mainstay of the treatment. None of the participants had received IS treatment before a renal histological confirmation.

### Renal histopathology

All kidney tissue specimens were obtained by percutaneous kidney biopsy, and examined using light microscope, immunofluorescent, and electron microscope. All histological slides were evaluated by an experienced renal pathologist. The four pathologic variables of the Oxford classification, which was used in this study, were scored as follows: mesangial score ≤0.5 (M0) or >0.5 (M1), absence (S0) or presence (S1) of segmental glomerulosclerosis, absence (E0) or presence (E1) of endocapillary hypercellularity, and tubular atrophy/interstitial fibrosis that was graded as T0 (≤25 %), T1 (26–50 %) or T2 (>50 %) [29].

### Measurement of the 25-hydroxyvitamin D

Serum 25(OH)D was measured using electrochemiluminescent immunoassay (ECLIA) with a Roche Elecsys 10100/201 system (Roche Diagnosis Elecsys) according to the manufacturer’s protocol. All measurements were performed in a blind manner and in duplicate. Vitamin D deficiency is defined as 25-hydroxy-vitamin D < 15 ng/ml.

### Statistical analyses

Descriptive data were analyzed for all variables. For continuous variables, results were presented as mean ± SD. Variables were compared using one-way analysis of variance (ANOVA) and unpaired Student’s *t*-test for normally distributed variables whereas Kruskal-Wallis Test was used for non-normally distributed variables. Categorical variables were recorded as frequency counts, and intergroup comparisons were analyzed using a chi-squared test. Event free survival curves were derived from the Kaplan-Meier method and differences between the two curves were tested using the logrank test. The model of the Cox proportional hazards was used to identify independent predictors for the development of the primary endpoint. A correlation analysis was conducted in order to avoid multi-collinearity; only one variable in highly correlated variable sets was selected for multivariate analysis. Statistically significant covariables from univariate analysis and clinically important covariables were included in the final multivariate Cox proportional hazard regression analysis, and backward elimination and stepwise selection approaches were conducted. A P < 0.05 is considered statistical significance. Data analysis was performed using the SPSS for Windows, version 12.0 (SPSS, Chicago, IL, USA).

## Results

### Basic clinical information of the participants

Table 1 shows the clinical characteristics of our study population (*n* = 105) at the time of kidney biopsy. The median age was 34 years old, and 54.3 % of the participants were men. Average level of the systolic BP, proteinuria, and baseline eGFR were 131 mmHg, 2.04 g/24 h, and 75.46 mL/min/1.73 m^2^, respectively. One hundred and one patients (96.2 %) had 25 (OH) vitamin D levels < 30 ng/ml, and fifty-one patients (48.6.5 %) had 25 (OH) vitamin D < 15 ng/ml, which are the thresholds for 25 (OH) vitamin D insufficiency and deficiency, respectively. By CDK stage, the proportions of patients with 25(OH)D <15 ng/ml were:55 % (22 of 40) at stage 1,42.9 % (12 of 28) at stage 2,61.9 % (13 of 21) at stage 3,83.3 % (5 of 6) at stage 4, and 90 % (9 of 10) at stage 5(shown as Table 2). A total of 83 (79 %) patients were treated with RAS blockers at the time of the kidney biopsy and 38 (36.2 %) patients received immunosuppressive therapy within 2–4 weeks after the kidney biopsy.

**Table 1:** Baseline characteristics of the study group with IgAN

	Total (*n* = 105)
Age (year)	34.73 ± 12.19
Male (n/%)	57(54.3 %)
Smoker (n/%)	32(30.5 %)
Body mass index (kg/m^2^)	23.2 ± 3.18
Systolic blood pressure (mmHg)	131.21 ± 23.43
Diastolic blood pressure (mmHg)	81.57 ± 16.84
Microscopic hematuria (n/%)	67 (63.8 %)
Proteinuria(g/24 h)	2.04 ± 1.09
eGFR (mL/min/1.73 m^2^)	75.45 ± 39.18
Serum albumin (mg/dL)	33.88 ± 7.91
Serum total cholesterol (mg/dL)	5.76 ± 2.09
Serum IgA (mg/dL)	2.97 ± 1.92
Uric acid (mg/dL)	404.81 ± 123.59
Medical treatment (n/%)	
RAS blockers	83 (79 %)
Immunosuppressant	38 (36.2 %)

Data are presented as mean ± SD. eGFR, estimated glomerular filtration rate; IgAN, IgA nephropathy; RAS, renin-angiotensin system

**Table 2:** The correlation between clinical parameters and 25(OH)D level at the time of kidney biopsy

Variables	Serum 25-hydroxyvitamin (ng/mL)		*P*-value
	≥15 ng/mL	<15 ng/mL	
Age (years)	36.19 ± 10.86	32.87 ± 13.62	0.18
Body mass index (kg/m^2^)	23.05 ± 2.86	23.39 ± 3.57	0.59
Systolic blood pressure (mmHg)	123.95 ± 17.26	140.52 ± 26.96	0.001
Diastolic blood pressure (mmHg)	76.66 ± 14.47	87.87 ± 17.70	0.001
Proteinuria(g/24 h)	1.23 ± 1.27	3.07 ± 2.06	<0.001
eGFR (mL/min/1.73 m^2^)	83.42 ± 32.64	65.24 ± 44.56	0.023
CKD stage 1(n/%)	18 (45 %)	22 (55 %)	0.069
CKD stage 2(n/%)	16 (57.1 %)	12 (42.9 %)	
CKD stage 3(n/%)	8 (38.1 %)	13 (61.9 %)	
CKD stage 4(n/%)	1 (16.7 %)	5 (83.3 %)	
CKD stage 5(n/%)	1 (10 %)	9 (90 %)	
Serum albumin (mg/dL)	38.08 ± 5.02	28.50 ± 7.72	<0.001
Serum total cholesterol (mg/dL)	5.14 ± 1.21	6.57 ± 2.64	<0.001
Serum IgA (mg/dL)	3.15 ± 2.24	2.73 ± 1.38	0.27
Uric acid (mg/dL)	374.31 ± 112.68	443.93 ± 127.11	0.004

eGFR, estimated glomerular filtration rate

### The correlation between clinical parameters and 25(OH)D

**Fig. 1:**
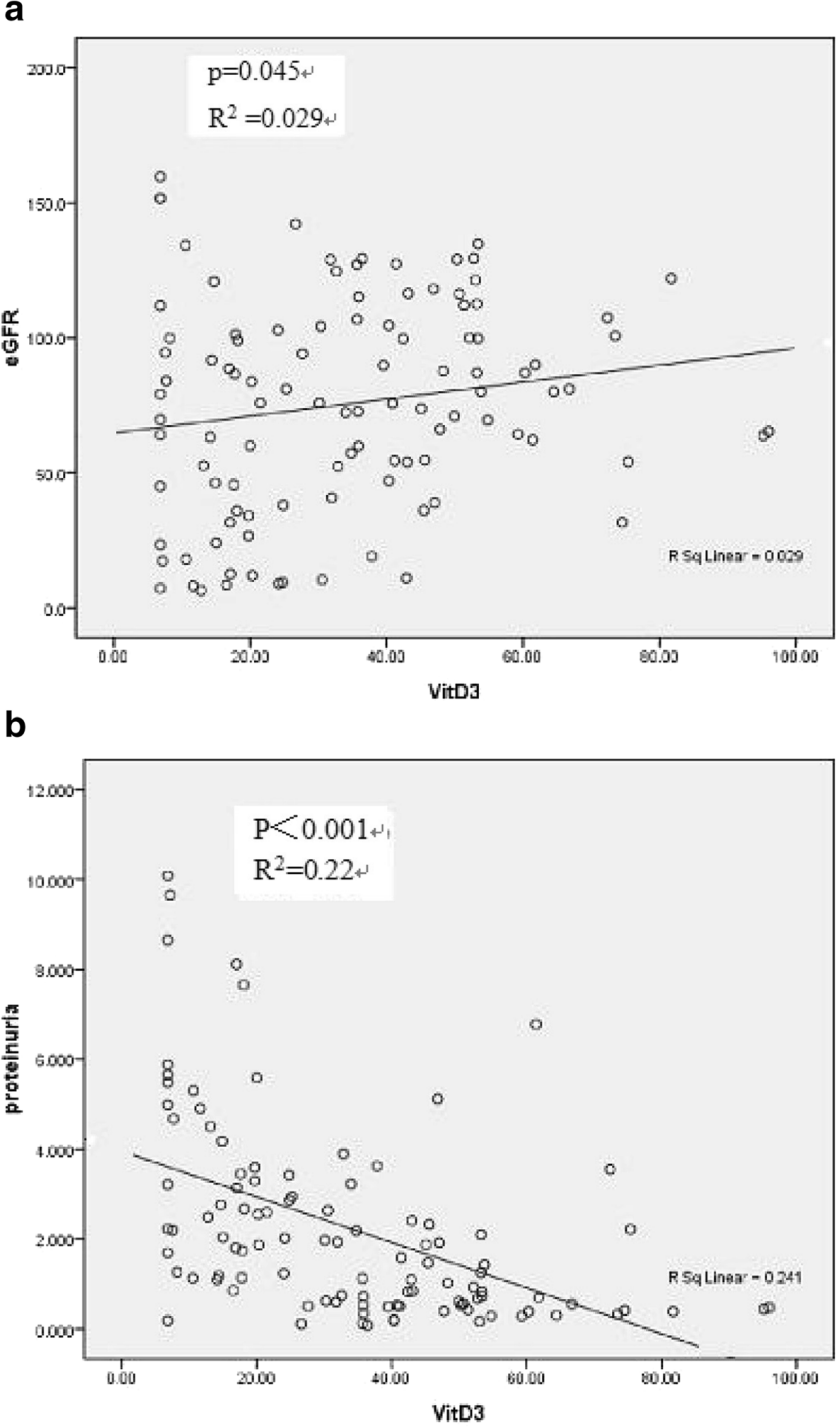
The 25(OH)D level is correlated negatively with eGFR (**a**) and positively with proteinuria (**b**), respectively, at the time of kidney biopsy

To identify the relationship between the serum level of 25(OH)D and several clinical parameters, we dichotomized the patients, based on their serum concentration of 25(OH)D, into two groups: levels of 25(OH)D < 15 ng/ml vs 15 ng/ml or greater. Compared with those showing a higher 25(OH)D level, those with a 25(OH)D deficiency were significantly associated with a lower eGFR and a higher proteinuria level (Table 2). Age, BMI and serum IgA level were not correlated with the 25(OH)D concentration. Stepwise increases in the uric acid level and BP but a decrease in the serum albumin level were also associated with the 25(OH)D level. These correlations were observed as well when the 25(OH)D level was used as continuous variables (Table 3 and Fig. 1). There was a significant positive correlation between the 25(OH)D level and the eGFR (*r* = 0.196, *P* < 0.05). Proteinuria was inversely correlated with the 25(OH)D level (*r* = -0.553, *P* < 0.01). We also conducted a correlation analysis between serum phosphorus, calcium, and PTH levels with 25(OH)D level (result shown in Additional file 1).

**Table 3:** Spearman correlation coefficients between various clinical parameters in IgAN patients

	proteinuria	25(OH)D	SBP	ALB	UA
eGFR	−0.377**	0.196*	−0.656**	0.155	−0.513**
proteinuria	1	−0.553**	0.464**	−0.564**	0.219*
25(OH)D		1	−0.316**	0.665**	−0.140
SBP			1	−0.272**	0.302***
ALB				1	−0.065
UA					1

*eGFR*, estimated glomerularfiltration rate, *SBP*, systolic blood pressure, *ALB* albumin, *UA* uric acid

* *P* < 0.05, **P < 0.01, ***P < 0.001

### Relationship between histologic features and the plasma 25(OH)D

We examined the association between the plasma 25(OH)D level at the time of initial diagnosis and the severity of histologic lesions, and found that the plasma 25(OH)D level was associated with the percentage of interstitial fibrosis/tubular atrophy. A lower plasma 25(OH)D level indicated a higher tubulointerstitial score with the aforementioned Oxford classification (*P* = 0.008; Table 4).

**Table 4:** Plasma 25(OH)D level associated with tubular atrophy/interstitial fibrosis in IgA nephropathy

Oxford score	25(OH)D level (ng/ml)	*P*
M0	14.55 ± 8.70	0.142
M1	12.10 ± 7.72	
E0	13.76 ± 8.50	0.443
E1	11.44 ± 4.19	
S0	11.79 ± 8.22	0.198
S1	15.17 ± 8.33	
T0	15.41 ± 8.89	0.008
T1	11.07 ± 6.80	
T2	8.84 ± 4.75	

### The impact of circulating 25(OH)D on clinical outcome

**Fig. 2:**
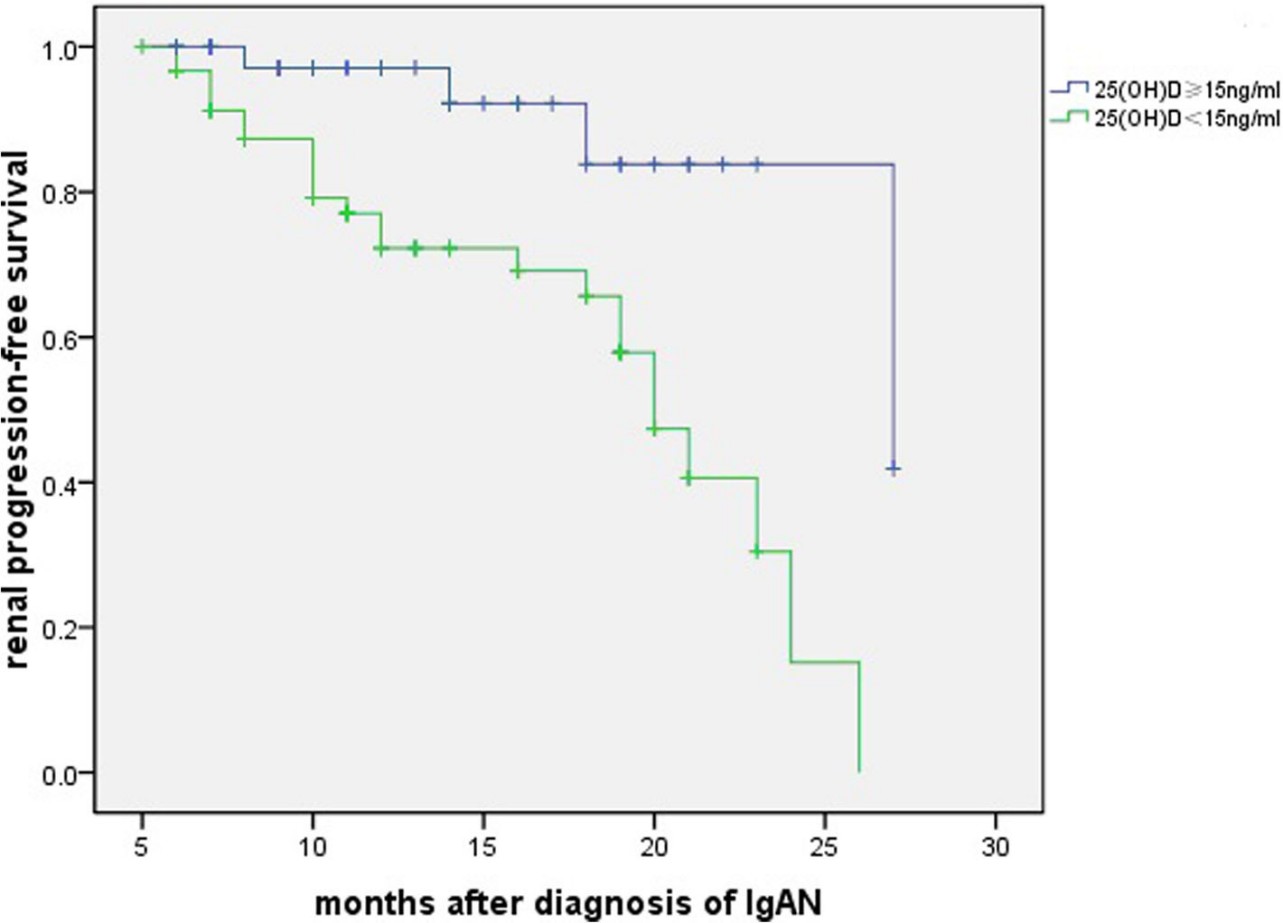
The patients with a deficiency of 25(OH)D are significantly associated with a higher risk for renal progression compared to those with a higher level of 25(OH)D

**Fig. 3:**
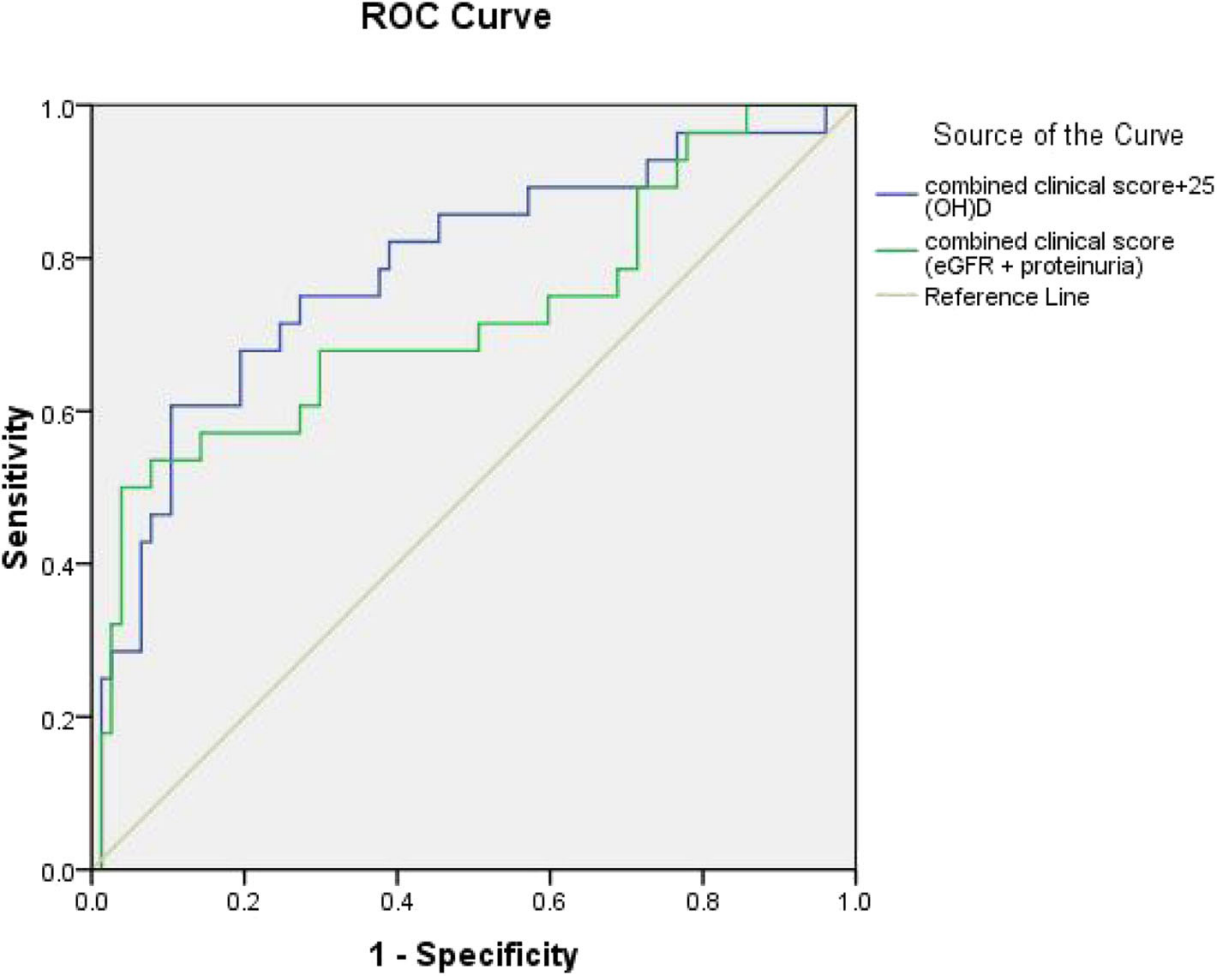
An ROC curve analysis with various biomarkers for renal progression showing that the circulating level of 25(OH)D add significant predictive information for patients’ renal progression

A total of 28 (26.7 %) patients reached the primary end-point (renal progression; eGFR declined by 30 % or more compared with the baseline level) during a median follow-up period of 13 months. The primary endpoint was reached by 24 patients with deficiency (39.3 %) and 4 patients without (9.1 %). Time-to-event analysis showed that those patients with a 25(OH)D deficiency showed a significantly higher risk for renal progression compared with their counterparts (Fig. 2). A Cox proportional hazards regression analysis revealed that 25(OH)D deficiency, is an independent risk factor for renal progression, after adjustment for age, gender, systolic BP, proteinuria, eGFR, subsequent vitamin D therapy, immunosuppressives treatment and ACEI/ARB treatment [HR 5.99, 95 % confidence intervals (CIs) 1.59–22.54, *P* = 0.008] (Table 5). Moreover, as shown in Fig. 3, the ROC curve was made comparing between combined clinical score (eGFR + proteinuria) vs combined clinical score + vitamin D level. It revealed that vitamin D can add significant predictive information to the renal progression, manifested as a larger area under the ROC curves (AUCs); the combined model (eGFR, proteinuria and vitamin D score), 0.785; combined clinical score, 0.722.

**Table 5:** Risk factors for renal progression in multivariate Cox regression analysis

	HR (95 % CI)	*P* value
Vitamin D deficiency	5.99 (1.59–22.54)	0.008
Male	1.87 (0.59–5.94)	0.287
eGFR	0.99 (0.97–1.01)	0.145
SBP	1.03 (1.00–1.05)	0.036
ALB	1.05 (0.99–1.12)	0.103
T0(reference)	1	
T1-2	2.76 (0.68–11.24)	0.156
Proteinuria	1.14 (0.82–1.57)	0.432

*ACEI* angiotensin converting enzyme inhibitor, *ARB* angiotensin II receptor blockade, *eGFR* estimated glomerularfiltration rate, *SBP*, systolic blood pressure, *ALB* albumin

## Discussion

Results from this study display several relationships between the plasma 25(OH)D level and several clinical parameters related to the IgAN histological damage and its severity. Our results also suggest that the plasma 25(OH)D level at the time of initial diagnosis may be an independent inverse-predictor of IgAN progression.

Vitamin D deficiency or insufficiency is highly prevalent among patients with CKD. An ethnically homogeneous cohort by Ravani et al. showed that the prevalence of a 25(OH)D deficiency or insufficiency was increased from 25 % in stage 2 to 56 % in stage 5 [8]. Cumulative evidence suggests an association of a low 25(OH)D level with clinical parameters related to a kidney damage. For example, Ravani et al. found that the baseline level of 25(OH)D was directly and significantly correlated with eGFR [8]. Diniz et al. also revealed an inverse correlation between the 25(OH)D level and proteinuria [30]. Furthermore, an inverse relationship between blood pressure and the 25(OH)D level has been documented as well in a number of epidemiological studies [31, 32]. Our observations are consistent with these previous reports.

Our observation of a more severe tubulointerstitial damage in patients with a 25(OH)D deficiency is of interest and suggests that a 25(OH)D deficiency in an early stage of IgAN may point to an advanced renal injury. Interstitial fibrosis together with tubular athrotrophy is a hallmark of chronic renal failure and strongly correlates with deterioration of renal function. Zhang Y. et al. [33] have established vitamin D receptor-null mice that allow a manipulation of unilateral ureteral obstruction for 7 days. Compared with the wild-type mice, the VDR-null mice develop more severe renal damage with marked tubular atrophy and interstitial fibrosis. Gonçalves et al. [34] found recently that a 25(OH)D deficiency might potentiate tubulointerstitial damages (fibrosis, inflammatory infiltration, tubular dilation and atrophy) through those inflammatory pathways that involve TGF-β1. Increased expression of TGF-β1 and decreased expression of VDR and Klotho protein are observed in VD deficient rats. The essential role of TGF-β1 and the protective effect of Klotho protein in pathogenesis of various renal diseases, including IgAN, have been documented in different clinical and experimental models [35–37].

There has been increasing evidence recommending the plasma 25(OH)D level as a good indicator for deterioration of renal function in various kidney diseases. In a prospective study with a cohort of predialysis patients with CKD, Nakano et al. [38] identified the serum 25(OH)D concentration as a significant predictor of renal composite outcome of doubling serum creatinine and ESRD. In the same cohort, Hamano et al. [39] found a nonlinear association between the serum 25(OH)D concentration and annual decline in the renal function. A meta-analysis of clinical observations reveals an inverse association between all-cause mortality and the serum 25(OH)D concentration [12], whereas our study shows that the circulating 25(OH)D level determined at the time of diagnosis predicts the renal progression. This predictability is not only independent of other important risk factors relevant to renal progression in IgAN but is also unaffected by the treatment of vitamin D, RAS blockers or immunosuppresors. Given that tubulointerstitial damage is one of the most important risk factors for renal progression in IgAN, our findings that connect the renal histologic features to the circulating level of 25(OH)D may partly explain why a decreased 25(OH)D level predicts the prognosis of IgAN.

In our study the follow-up period is too short to assess the long-term renal outcomes such as ESRD, and death in this patient population. Nevertheless, it is noteworthy that this is the first study suggesting a prognostic value of the circulating 25(OH)D in IgAN patients, although its prognostic value for the much longer outcome still awaits further study. Moreover, in the last decade observational studies have noted links between vitamin D deficiency and poor cardiovascular outcomes in CKD patients. Our study only demonstrated the reverse relationship of vitamin D and the blood pressure, but did not test the relationship of 25(OH)D with other cardiovascular outcomes such as incident coronary heart disease and heart failure. Given the high-cardiovascular risk of CKD, the issue deserves to be addressed in well powered future studies. Finally, our study did not address the vitamin D treatment effect, thus an adequately powered randomized controlled trial should be designed to determine whether 25 (OH) vitamin D therapy is able to slow the progression of IgAN.

## Conclusions

In conclusion, 25(OH)D deficiency is significantly correlated with a poorer renal function and more severe renal pathological features, and is strongly associated with increased risk of renal progression in IgAN patients, making a 25(OH)D deficiency a good prognostic marker to predict the severity and clinical outcome in IgAN patients. Therefore, determining the circulating level of 25(OH)D may be important and informative for the identification of high-risk patients and for the proper management of IgAN.

## Additional file

Additional file 1: A correlation analysis between serum phosphorus, calcium, and PTH levels with vit D levels. 25(OH) positively related with serum calcium and negatively related with serum phosphorus and PTH. (DOC 82 kb)

## Abbreviations

25(OH)D: 25-hydroxy-vitamin D
ACEI: Angiotensin converting enzyme inhibitor
ANOVA: One-way analysis of variance
ARB: Angiotensin II receptor blockade
BMI: Body mass index
CIs: Confidence intervals
CKD: Chronic kidney diseases
CKD-EPI: Chronic kidney disease epidemiology collaboration
eGFR: Estimated glomerular filtration rate
ESRD: End-stage renal diseases
IgAN: IgA nephropathy
IS: Immunosuppressors
RAS: Renin-angiotensin system
