# Supplementary figures and images for: Vitamin D deficiency may predict a poorer outcome of IgA nephropathy
Source: BMC Nephrol. 2016 Nov 2;17:164. doi: 10.1186/s12882-016-0378-4 (PMC5094030; doi:10.1186/s12882-016-0378-4)

A correlation analysis between serum phosphorus, calcium, and PTH levels with vit D levels.


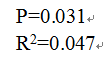

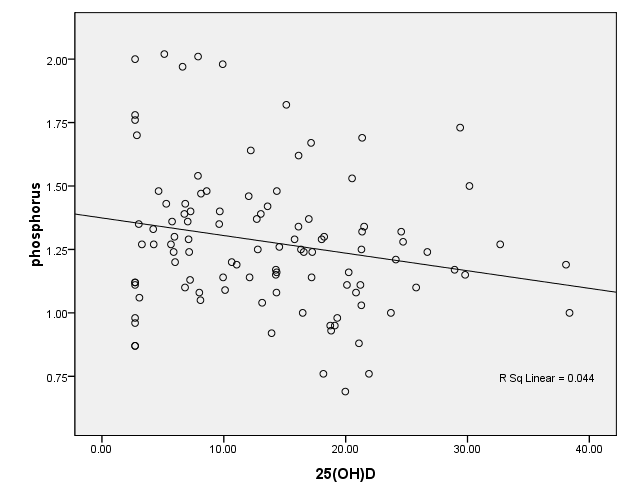


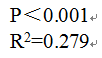

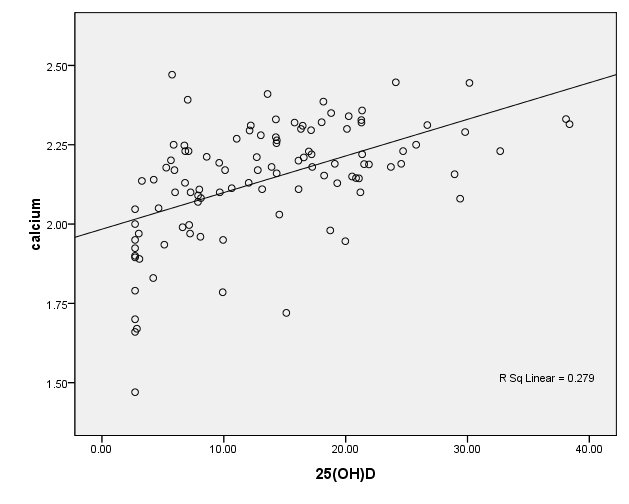


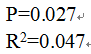

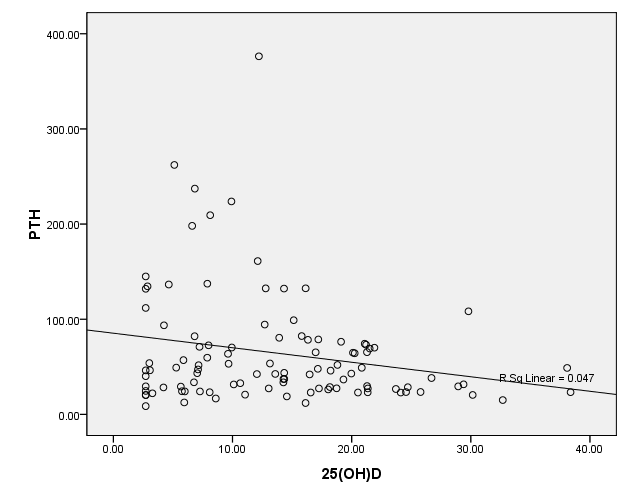

Supplement: Additional file 1: — A correlation analysis between serum phosphorus, calcium, and PTH levels with vit D levels. 25(OH) positively related with serum calcium and negatively related with serum phosphorus and PTH. (DOC 82 kb) [file 12882_2016_378_MOESM1_ESM.doc]
